# Synthesis of a Stimulus-Sensitive Copolymer with Response to Temperature and pH in Silicone Catheters

**DOI:** 10.3390/polym17233107

**Published:** 2025-11-23

**Authors:** Yanet González Alonso, Emilio Bucio

**Affiliations:** Departamento de Química de Radioquímica, Instituto de Ciencias Nucleares, Universidad Nacional Autónoma de México, Circuito Exterior, Ciudad Universitaria, Mexico City C.P. 04510, Mexico

**Keywords:** silicone catheter, N-vinylcaprolactam (NVCL), N-vinylimidazole (NVIM), graft, smart polymer, gamma radiation

## Abstract

This work aimed to synthesize a graft copolymer with temperature- and pH-responsive properties. The synthesis of SR-cat-*g*-(NVCL/NVIM) was carried out using the direct irradiation method with a ^60^Co gamma-ray source (Gammabeam 651 PT). The temperature- and pH-responsive monomers, N-vinylcaprolactam (NVCL) and N-vinylimidazole (NVIM), respectively, were grafted onto silicone catheters. The effects of irradiation dose and monomer concentration on grafting efficiency were studied. A direct relationship was found between grafting efficiency and both irradiation dose and monomer concentration. As these parameters increased, the grafting percentage also increased. The biomaterial was characterized by using Fourier-Transform Infrared Spectroscopy (FT-IR), Differential Scanning Calorimetry (DSC), Thermogravimetric Analysis (TGA), Scanning Electron Microscopy (SEM), swelling, and a water contact angle measurement. The swelling behavior was also investigated by varying temperature and pH. The Lower Critical Solution Temperature (LCST) was observed around 34 °C, and pH sensitivity was detected between pH 8 and 8.5. Mechanical tests were performed to conduct a systematic analysis relating to the grafting percentage and the ratio between grafted polymers and Young’s modulus. Finally, the loading and release capacity of norfloxacin in the modified catheters was evaluated.

## 1. Introduction

Healthcare-associated infections occur due to the use of temporary or permanent medical devices, posing a significant threat to patient health and carrying a high risk of infection [1]. Many medical devices are associated with infections, including breathing equipment, urinary catheters, probes, central venous catheters, pacemakers, and orthopedic prostheses. An example of this issue is urinary tract infections caused by biofilm formation on the surface of catheters [2]. These implants are made of synthetic polymers, which are highly hydrophobic and thus promote bacterial colonization [3].

Silicone is a synthetic polymer widely used as a biomaterial due to its biocompatibility, flexibility, elasticity, and chemical properties [4,5]. However, it has the drawback of being hydrophobic, which encourages the formation of microbial communities. Therefore, it is suggested that its structure be modified with smart polymers that respond to temperature and pH stimuli, thereby enhancing its properties for medical device applications and increasing its resistance to biofilm formation through the addition of hydrophilic groups. Smart polymers grafted with N-vinylcaprolactam (NVCL) and N-vinylimidazole (NVIM) can respond to temperature [6] and pH [7], respectively. Moreover, NVCL is a biocompatible polymer [8], and NVIM can show antimicrobial properties [9,10]. The resulting copolymer from the grafting process exhibits a dual response, making it a highly promising material for biomedical applications.

Gamma radiation is a form of electromagnetic radiation characterized by the high energy of its photons. It is a widely used technique for altering polymeric materials. The main advantages of this method lie in its efficiency and control over the polymerization process, which can be achieved without the use of additives, making it a clean technique [11,12]. There are three primary methods: pre-irradiation, oxidative pre-irradiation, and direct method [13]. The direct method, in a single step, is a quick and straightforward process in which the polymer matrix and monomers are irradiated simultaneously, forming free radicals that initiate polymerization [14].

Previous studies have reported the grafting of N-vinylcaprolactam and N-vinylimidazole onto silicone films using the direct method and the characterization of the resulting copolymer, demonstrating that gamma radiation modification is an effective technique for obtaining stimulus-responsive polymers [15]. However, mechanical testing, scanning electron microscopy analysis, and water contact angle analysis were not included, and the drug loading and release capacity of the biomaterial were not evaluated. NVCL and NVIM graft polymerization in polypropylene films have also been explored using direct and pre-oxidative methods with gamma radiation, and the influence of various parameters on the grafting has been analyzed [16].

In this study, silicone catheters were modified by grafting NVCL and NVIM monomers through gamma irradiation using a one-step direct method. The effects of irradiation dose and monomer concentration on the grafting percentage were examined. The graft copolymer was characterized by using FTIR-ATR, TGA, DSC, swelling, and SEM. Additionally, physicochemical properties were analyzed, and the responses to pH and temperature were assessed. Finally, the capacity for loading and releasing Norfloxacin in the pH- and temperature-sensitive copolymer was evaluated.

## 2. Materials and Methods

### 2.1. Reagents and Solvents

The silicone catheters (SR-cat) with a 5 mm diameter, 2 mm wall thickness, and 2.5 cm length were obtained from Degania Silicone (Degania Bet, Israel). The precursor monomers, N-vinylcaprolactam and N-vinylimidazole, were sourced from Sigma-Aldrich Co. (St. Louis, MO, USA) and purified by reduced-pressure vacuum distillation. The solvents toluene and ethanol were supplied by Sigma Aldrich Co. (St. Louis, MO, USA) and CONQUIMEX (Ecatepec, Mexico). The cobalt-60 source (^60^Co Gammabeam 651-PT, MDS Nordion, Ottawa, ON, Canada) was provided by the Institute of Nuclear Sciences, UNAM.

### 2.2. Synthesis of SR-cat-g-(NVCL/NVIM) by the Direct Method

**Figure 1:**
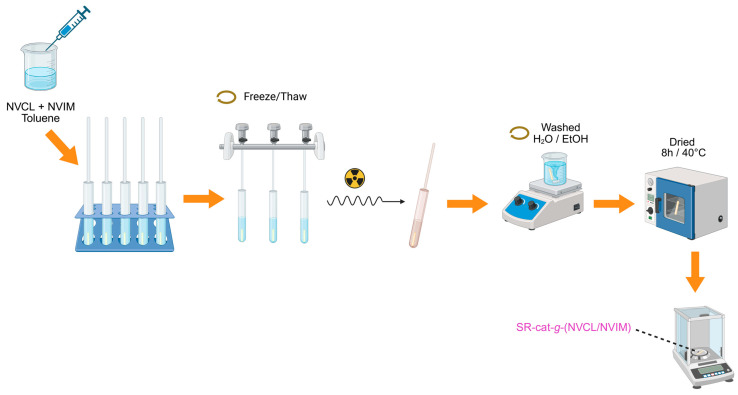
Methodology for the synthesis of SR-cat-*g*-(NVCL/NVIM).

The synthesis of SR-cat-*g*-(NVCL/NVIM) was performed by direct irradiation method in a single step using a gamma-ray source (^60^Co). The SR-cat samples, each 2.5 cm long, were washed with ethanol for 24 h to remove impurities (the ethanol was changed twice), vacuum-dried, and placed in glass tubes to prepare ampoules for irradiation. The NVCL monomer was added first, and then the NVIM monomer. Equimolar amounts of NVCL/NVIM monomers and toluene solvent were added, totaling 6 mL. Oxygen was removed from the ampoules using five freeze–thaw cycles in a vacuum line cooled with liquid nitrogen, and the ampoules were then sealed for irradiation. After grafting, the catheters were washed with two cycles of water for three hours each, and one cycle of ethanol for three hours. Finally, the samples were dried for approximately eight hours at 40 °C in a vacuum oven and finally weighed. This process was repeated three times. Figure 1 illustrates the methodology used to synthesize SR-cat-*g*-(NVCL/NVIM). The graft percentage was calculated using Equation (1):

(1) $$Grafting\;\left(\%\right)=\;\frac{Wf-Wi}{Wi}\;\times \;100$$

where:

Wf*:* final weight of the grafted copolymer.

Wi*:* initial weight of the SR-cat.

#### 2.2.1. Effect of Dose

The effect of irradiation dose on graft yield was studied at a fixed monomer and solvent concentration of 50 (%*v*/*v*). The SR-cat samples were irradiated at 20, 30, 40, 50, 60, and 70 kGy, with a dose rate of approximately 7.6 kGy/h.

#### 2.2.2. Effect of NVCL/NVIM Concentration

The SR-cat samples were irradiated at a fixed dose of 50 kGy, and the monomer concentration of NVCL/NVIM monomers varied: 5, 10, 20, 30, 40, and 60% (*v*/*v*) in a toluene solution.

### 2.3. Swelling Tests

The swelling behavior of SR-cat-*g*-(NVCL/NVIM) was examined over time, from 15 min to 24 h. Four dry samples with varying graft percentages and a control were weighed. The method involved immersing the samples in distilled water, removing them at specific times, draining excess water, and reweighing them. This process was repeated in triplicate.

Swelling was also studied in phosphate buffer solutions with pH values ranging from 2 to 12 at 25 °C to examine the pH response of SR-cat-*g*-(NVCL/NVIM). The procedure was followed as described above.

The swelling percentage was calculated using Equation (2).

(2) $$Swelling\;\left(\%\right)=\frac{\left(W_{2}-W_{1}\right)}{W_{1}}\times 100$$

where

*W*_1_*:* weight of the dry sample.

*W*_2_*:* final weight of the swollen sample.

### 2.4. LCST Measured by DSC

A sample of SR-cat-*g*-(NVCL/NVIM) 101% graft (7.6 mg) was taken and swollen in distilled water overnight. It was encapsulated in an aluminum tray. This pellet was analyzed using a TA Instruments 2010 differential scanning calorimeter (DSC), (TA Instruments New Castle, DE, USA), alongside a reference sample (an empty tray), at a heating rate of 1 °C/min from 10 to 90 °C.

### 2.5. Water Contact Angle

The samples were flattened with glass plates and dried in a vacuum oven for 8 h at 40 °C. This analysis involved placing a drop of distilled water on the flat catheter surface and measuring the contact angle between the drop and the surface. This process was repeated four times on both the inner and outer surfaces of the catheter. The equipment used was a DSA 100 Krüss GMbH goniometer (Krüss, Hamburg, Germany).

### 2.6. Infrared Spectroscopy

The unmodified silicone catheter, the NVCL/NVIM copolymer, and the modified catheters were examined using FTIR spectroscopy with a Perkin-Elmer Spectrum 100 spectrophotometer (Perkin-Elmer Cetus Instruments, Norwalk, CT, USA). The analysis involved 16 scans over a frequency range from 4000.00 to 650.00 cm^−1^, with a resolution of 4.00 cm^−1^ in the ATR module.

### 2.7. Thermogravimetry

TA Instruments USA TGA Q50 (TA Instruments, New Castle, DE, USA) was used, with approximately 10 mg of the sample placed in the instrument tray. An inert nitrogen atmosphere was used in a temperature range of 25 to 800 °C with a heating rate of 10 °C/min.

### 2.8. Differential Scanning Calorimetry

Approximately 5 to 10 mg of the samples were weighed and pressed into a capsule within an aluminum tray. The sealed samples were then analyzed using a TA Instruments 2010 differential scanning calorimeter (DSC) in the USA, alongside a reference sample (empty tray). The analysis was conducted at a heating rate of 10 °C/min from 25 to 400 °C under a nitrogen atmosphere.

### 2.9. Scanning Electron Microscopy

This analysis was performed using a JEOL 5900-LV (JEOL, Tokyo, Japan) instrument in Japan under low-vacuum conditions, with gold coating and backscattered electron detectors. Images were captured at various magnifications, and both surface and cross-sectional observations were made. Additionally, surface elemental analysis was conducted with SEM-EDS.

### 2.10. Mechanical Tests

The tensile test was conducted on approximately 30 mm test specimens using Shimadzu universal testing equipment (Shimadzu, Kyoto, Japan), model AGS-X, from Tokyo, Japan, with a crosshead speed of 10 mm/min and a jaw separation of 10 mm.

### 2.11. Drug Loading and Release

#### 2.11.1. Norfloxacin Loading

Samples of approximately 95 mg of SR-cat-g-(NVCL/NVIM) and the unmodified silicone were taken. Eight milliliters of the 8.0 µg/mL norfloxacin solution were added to the amber vials containing the samples, which were then placed in a water bath at 25 °C. Using UV-Vis spectroscopy (P3 Mapada Instruments, Shanghai, China) at 268 nm, the loading was monitored over 148 h. Calibration curves were prepared to quantify the amount of drug loaded. All these procedures were performed in triplicate. The amount of drug loaded was calculated using Equation (3).

(3) $$Drug\;loaded\;\left(\frac{mg}{g}\right)=\frac{Ci-Cf}{W}\times V$$

where

Ci y Cf: initial and final concentration.

W: weight of the samples.

V: volume of the solution.

Calibration Curve for Loading:

Several aliquots of the norfloxacin solution were taken to prepare solutions with concentrations of 1, 2, 3, 4, 5, 6, 7, and 8 µg/mL, which were then measured with distilled water. Monitoring was performed at 268 nm. The calibration curves were plotted using the obtained values. This process was performed in triplicate.

#### 2.11.2. Norfloxacin Release

The amber vials containing the loaded samples were added to 8 mL of phosphate-buffered saline (PBS) at pH 7.4. They were placed in a water bath with agitation at 100 rpm and 37 °C. The release over time was monitored using UV-Vis spectroscopy at 268 nm. Calibration curves were prepared to quantify the amount of drug released. All these procedures were performed in triplicate. The amount of drug released was calculated using Equation (4).

(4) $$Drug\;release\;\left(\frac{mg}{g}\right)=\frac{Cf}{W}\times V$$

where

Cf: final concentration.

W: weight of the samples.

V: volume of the solution.

Calibration Curve for Release:

Several aliquots were taken from the norfloxacin solution and mixed with PBS solution to prepare solutions with concentrations of 0, 0.02, 0.10, 0.25, 0.50, 1.00, 1.50, 2.00, 3.00, and 4.00 µg/mL. Monitoring was performed at 268 nm, using PBS as the blank. The corresponding calibration curves were created using the obtained values. This process was performed in triplicate.

## 3. Results

### 3.1. Synthesis of SR-cat-g-(NVCL/NVIM) by the Direct Method

The synthesis of SR-cat-*g*-(NVCL/NVIM) was carried out using the direct irradiation method with a ^60^Co gamma-ray source and toluene as the solvent, which promotes mass grafting and high yields. Temperature- and pH-responsive N-vinylcaprolactam and N-vinylimidazole monomers were grafted onto silicone catheters in a single-step process to modify their surface and create a new intelligent, hydrophilic biomaterial that responds to temperature and pH.

**Figure 2:**
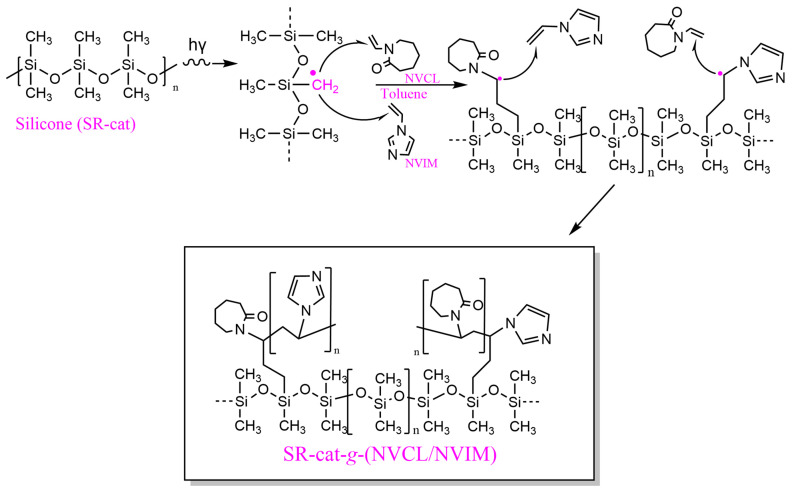
Proposed reaction mechanism for the formation of SR-cat-*g*-(NVCL/NVIM).

In the direct method, the silicone polymer matrix, along with NVCL and NVIM, is irradiated simultaneously, allowing the formation of free radicals in silicone and/or the monomers. Radiation exposure creates four potential active sites in silicone (Figure S1): two from the excitation of side groups, and two from the breakage of the main chain, with the methyl radical being the most common [17,18,19]. The polymerization mechanism is illustrated in Figure 2, where the initiation step involves the formation of the primary radical of the methyl group (˙CH_2_). During propagation, the radical attacks the olefin of NVCL or NVIM, resulting in the formation of a second radical after the double bond breaks. The addition reaction terminates when the secondary radical reacts with the terminal part of another NVIM or NVCL molecule, forming the alternating copolymer SR-cat-g-(NVCL/NVIM).

#### 3.1.1. Effect of Dose

The effect of irradiation dose on graft yield was examined within a range of 20 to 70 kGy at a dose rate of 7.7 kGy/h. The synthesis was performed using a 50% monomer concentration ratio (NVCL/NVIM) in toluene.

**Figure 3:**
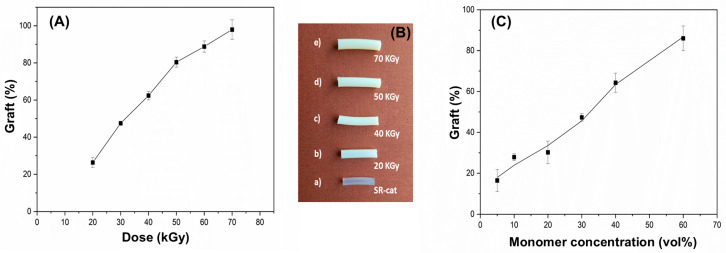
(**A**) Grafting of NVCL/NVIM onto SR-cat (SR-cat-*g*-(NVCL/NVIM) as a function of irradiation dose (monomers concentration 50 vol%); (**B**) Silicone catheters with different grafting degrees of: (a) 0%, (b) 26%, (c) 63%, (d) 80%, (e) 98%; (**C**) Grafting of NVCL/NVIM onto SR-cat (SR-cat-*g*-(NVCL/NVIM) as a function of monomers concentration at an irradiation dose of 50 kGy.

Figure 3A shows efficient grafting of NVCL and NVIM onto the silicone matrix, with a linear trend observed as the graft percentage increases with higher irradiation doses, reaching an average maximum of approximately 98%. This occurs because greater exposure to gamma radiation creates more active sites, resulting in more free radicals during the reaction. A noticeable change in the appearance of the grafted catheters was observed compared with the ungrafted silicone catheter. As irradiation and graft percentage increase, the catheters become harder and lose their characteristic transparency. Additionally, the grafted catheters grow larger in size and thickness due to mass grafting.

#### 3.1.2. Effect of NVCL/NVIM Concentration

The influence of monomer concentration on grafting yield was examined across 5, 10, 20, 30, 40, and 60%, at a dose rate of 7.4 kGy/h and an irradiation dose of 50 kGy.

Figure 3B shows that monomer concentrations from 5 to 30% result in yields below 50%, while concentrations of 40 to 60% yield higher results, reaching approximately 86% grafting. It was also observed that materials with low monomer concentrations were more fragile and broke when removed from the ampoule. Conversely, at a 60% monomer concentration, a rough surface was seen on the biomaterial.

### 3.2. Swelling Tests

**Figure 4:**
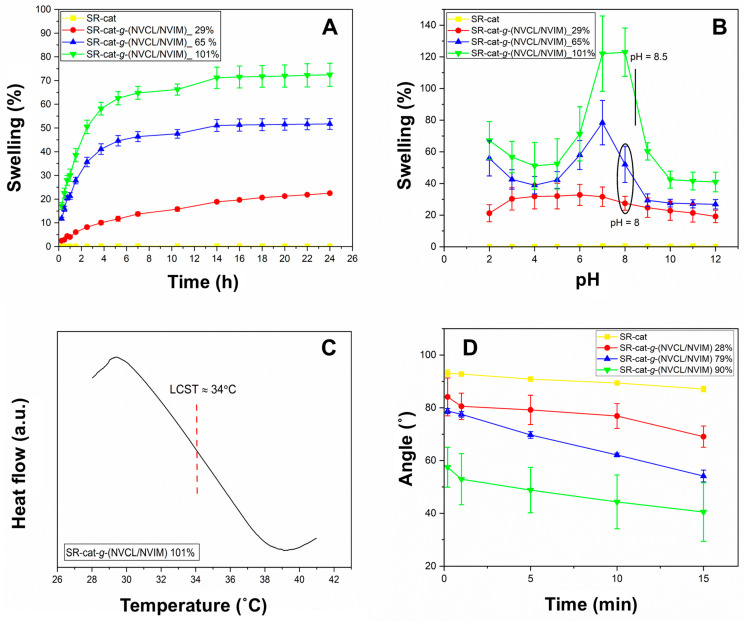
(**A**) Swelling limit of the different grafts in water (at 25 °C) as a function of time. (**B**) Swelling limit of the different grafts in PBS solutions at pH 2–12 and 25 °C. (**C**) DSC thermogram of water-swollen. (**D**) Contact angle behavior 0, 1, 5, 10, and 15 min.

The swelling behavior of SR-cat-*g*-(NVCL/NVIM) over time was examined from 15 min to 24 h (Figure 4A). Water absorption was rapid during the first 5 h, reaching a swelling limit at 14 h for all samples except the control silicone catheter, which did not swell because of its hydrophobic nature. A correlation was observed between graft percentage and degree of swelling: as the graft percentage increased, water absorption capacity also increased. This is due to the increase in hydrophilic groups in the material resulting from monomer grafting. These groups, such as the carbonyl (C=O) of the lactam ring and the nitrogen atoms of the imidazole ring, can form hydrogen bonds with water molecules.

### 3.3. pH-Sensitive

Critical pH is a characteristic of pH-sensitive polymers. Figure 4B illustrates the equilibrium swelling behavior as a function of pH (2 to 12) at 25 °C. As observed, the SR-cat sample does not display this property. The pH sensitivity is due to the imidazole group; this group can protonate or deprotonate depending on the pH of the medium. At acidic pH, the tertiary amine is protonated and behaves as a weak base, whereas at basic pH it is deprotonated, reducing its interaction with water molecules. Absorption varied with different grafting percentages, and swelling changed dramatically around pH 8, which is close to the pKa of the imidazole group. The shift in the critical pH of the samples falls within the range of 8 to 8.5. This can be explained by the presence of the aliphatic ring of NVCL, which has both hydrophilic and hydrophobic parts; the hydrophobic segment can influence the pH sensitivity of NVIM. Additionally, the literature indicates that the shift in the critical pH to alkaline values is due to the presence of some aliphatic chains or fragments attached to a pH-sensitive segment [20,21].

### 3.4. LCST Measured by DSC

To observe the LCST, DSC analysis was performed on the sample SR-cat-*g*-(NVCL/NVIM) with 101% graft. Figure 4C shows the thermogram of the copolymer, which was swollen in distilled water before analysis. To determine the LCST, the starting point of the endothermic peak was taken as the intersection of two tangent lines to the baseline, determined by the slope of the endothermic peak. A thermal transition attributed to the LCST is observed, occurring at 34 °C. The LCST value obtained matches the expected value and is close to physiological temperature, making it a potential system for drug-loading and release applications. The LCST of 34 °C enables precise release upon reaching body temperature and minimizes premature release, optimizing efficacy and safety in biomedical applications.

Stetsyshyn et al. propose that the presence of a critical solubility temperature in polymers is governed by interactions between the polymer and the solvent, by thermodynamic parameters of the mixture, and by hydrogen bonding. They establish that, at temperatures below the LCST, hydrophilic interactions between the polymer and solvent molecules predominate. When the temperature exceeds the LCST, hydrophobic interactions within the polymer chains become dominant, and weak crosslinking structures formed by hydrogen bonds between a water molecule and two oxygen atoms of adjacent lactam rings are observed [22].

### 3.5. Water Contact Angle

This analysis was conducted to evaluate the material’s hydrophilicity by placing water on its surface. The contact angle formed between a solid surface and a water droplet depends on the liquid’s surface tension and the surface’s properties.

In Figure 4D, the control silicone shows angles greater than 90°, confirming its hydrophobic nature. In contrast, the grafted catheters have angles less than 90°, indicating they are hydrophilic, as mentioned earlier. The higher the graft percentage, the more hydrophilic the material becomes, resulting in lower contact angles. This is because the material’s increased hydrophilicity allows it to form hydrogen bonds with water molecules. The order of increasing hydrophilicity is SR-cat < SR-cat-*g*-(NVCL/NVIM) 28% < SR-cat-*g*-(NVCL/NVIM) 79% < SR-cat-*g*-(NVCL/NVIM) 90% (Table S1). These findings agree with the results obtained in the limit swellings in Section 3.2.

### 3.6. Infrared Spectroscopy

**Figure 5:**
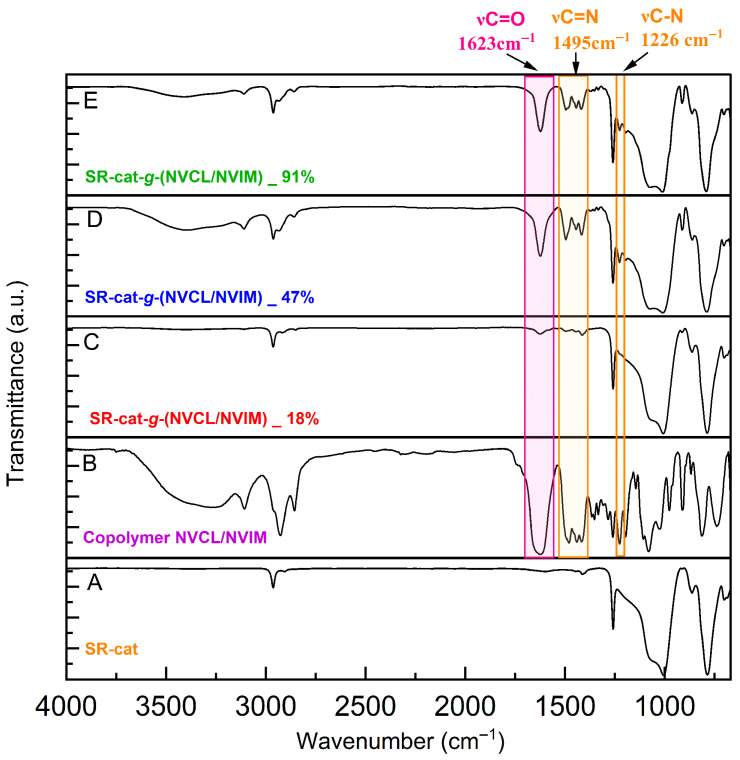
Comparison of FT-IR spectra of NVCL/NVIM copolymer, control catheter, and modified catheter with different graft yields.

The unmodified silicone catheter displayed bands corresponding to the asymmetric stretching of the Csp^3^-H of the CH_3_ groups at 2963 cm^−1^ and the bending vibrations (δ) at 1412 cm^−1^. It also showed characteristic bands of the siloxane group, with the Si-C stretching band at 1286 cm^−1^ and the Si-O-Si stretching band at 1006 cm^−1^ (Figure 5A) [23].

The SR-cat-g-(NVCL/NVIM) spectra at different grafting percentages (18, 47, and 91%) and the signals characteristic of the silicone catheter showed additional bands, with greater intensity in the D and E spectra (Figure 5). At approximately 2963 cm^−1^, corresponding to the C-H stretching; at around 1623 cm^−1^, related to the carbonyl (C=O) stretching; and at about 1495 cm^−1^, associated with the C=N stretching, there is a band at approximately 1444 cm^−1^ corresponding to the asymmetric and symmetric bending vibrations of CH_3_ and CH_2_ (δ^as^ + δ^s^); additionally, the band at about 1226 cm^−1^ indicates the C-N-C stretch. This confirms the grafting of the monomers, as the characteristic bands of NVCL and NVIM are present. In contrast, spectrum C (Figure 5) exhibits a band corresponding to the carbonyl and amine groups with low intensity. This likely results from the lower monomer grafting percentage on the catheter. These characteristic bands are confirmed in spectrum B (Figure 5), which corresponds to the NVCL/NVIM copolymer, where they appear with greater intensity.

### 3.7. Thermogravimetry

**Figure 6:**
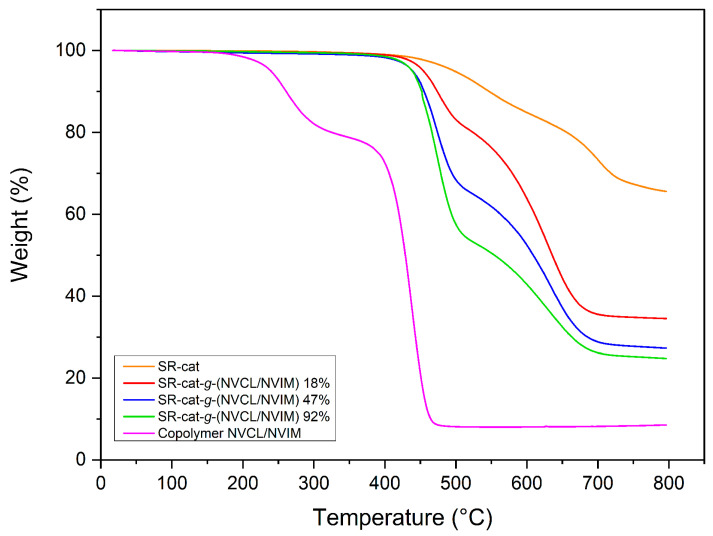
Comparative thermogram of unmodified silicone, silicone grafted at different percentages, and NVCL/NVIM copolymer.

Thermogravimetric analysis of the samples revealed their thermal stability. Samples of SR-cat, SR-cat-*g*-(NVCL/NVIM) with various grafting yields (18, 47, and 92%) and the NVCL/NVIM copolymer were compared; the results are shown in Figure 6. For characterization, an inert nitrogen atmosphere was used over a temperature range of 25 to 800 °C, at a heating rate of 10 °C min^−1^. The unmodified silicone catheter exhibited 10% weight loss at 547 °C, a decomposition temperature of 702 °C, and 66% residue at 800 °C, mainly consisting of SiO and SiO_2_. These findings confirm the high thermal stability of the silicone matrix.

The modified catheters exhibit similar behavior, with two distinct decomposition temperatures corresponding to two different stages of degradation. The 10% weight loss temperatures for SR-cat-*g*-(NVCL/NVIM)_18%, SR-cat-*g*-(NVCL/NVIM)_47%, and SR-cat-*g*-(NVCL/NVIM)_92% were observed at 474, 455, and 452 °C, respectively (Table S2). The first decomposition temperature corresponds to the loss of NVCL and NVIM monomer chains, while the second is due to the thermal breakdown of silicone [24,25,26]. The NVCL/NVIM copolymer exhibited a 10% weight loss at 261 °C. Two decomposition temperatures were identified: one at 262 °C, likely due to moisture absorbed from the environment, and another at 438 °C, related to the breakdown of NVCL and NVIM.VIM.

### 3.8. Differential Scanning Calorimetry

**Figure 7:**
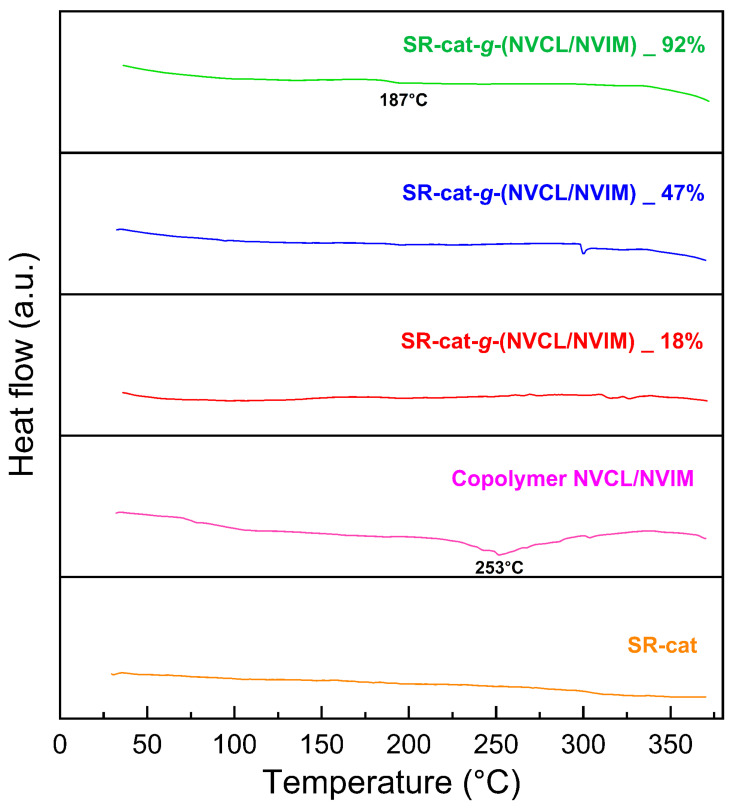
Thermogram of DSC analysis.

DSC characterization was conducted to determine the thermal transitions of the studied materials. This analysis helps us understand how polymeric materials behave as temperature increases; for this purpose, a reference sample is used. The control silicone specimens, silicone modified with different graft percentages, and the NVCL/NVIM copolymer were examined. Figure 7 displays the thermogram from the DSC analysis; for SR-cat, no transition is observed because it is an amorphous polymer.

Its typical second-order transition behavior marks the glass transition temperature (Tg) and depends on the heating or cooling rate. For copolymers, the Tg generally falls within the range of their homopolymers. The presence of a copolymer can interfere with molecular packing, leading to a reduction in interchain attraction forces; as a result, the predicted Tg may be higher than the actual Tg [27].

PolyNVCL is an amorphous polymer with a reported Tg close to 147 °C [28,29]. Meanwhile, PolyNIM has been reported to have a Tg in the range of 148–175 °C [30,31]. For SR-cat-*g*-(NVCL/NVIM) 18% and SR-cat-*g*-(NVCL/NVIM) 47%, a Tg would be expected; however, it is not observed in either case. For SR-cat-*g*-(NVCL/NVIM) 92%, which has the highest grafting, a transition is barely visible at approximately 187 °C, indicating a Tg. The thermogram shows that the NVCL/NVIM copolymer exhibits a thermal transition at 253 °C, which does not match the Tg reported for the NVCL and NVIM components. This behavior may be due to interactions among all the elements of the binary graft, leading to a shift in its value.

### 3.9. Scanning Electron Microscopy

Microscopy analysis was performed on SR-cat and SR-cat-*g*-(NVCL/NVIM) specimens with grafting percentages of 22, 62, and 112%. This study aimed to analyze the surface and cross-sections of the samples in the cut region. The conditions included gold coating, the use of backscattered electron detectors, and operation under vacuum.

**Figure 8:**
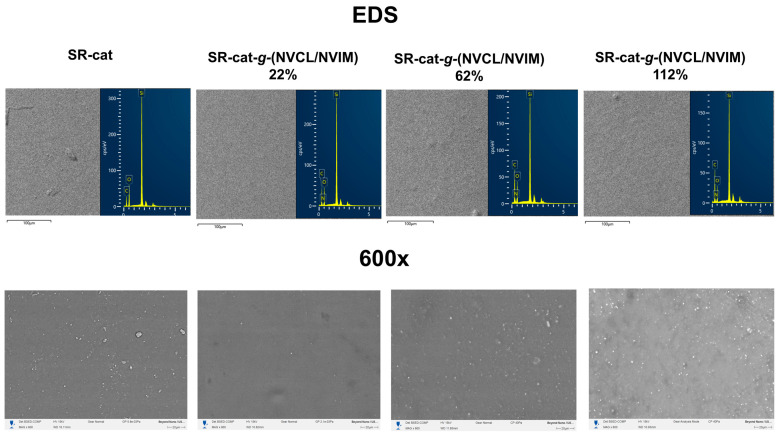
SEM micrographs of the surface of SR-cat, SR-cat-*g*-(NVCL/NVIM) 22%, SR-cat-*g*-(NVCL/NVIM) 62% and SR-cat-*g-*(NVCL/NVIM) 112%.

Figure 8 displays micrographs of the sample surfaces at 600× magnification and the EDS analysis, showing no significant differences in topography. Analysis of these images indicates bulk grafting, as the surface is uniform throughout the catheter. As mentioned in previous sections, using toluene as a solvent causes the silicone catheters to swell, promoting bulk grafting during polymerization. EDS analysis revealed an increase in carbon mass percentage of up to 14% in the grafted samples compared to unmodified silicone. Also, it detected nitrogen atoms in the modified catheters, which resulted from monomer grafting.

**Figure 9:**
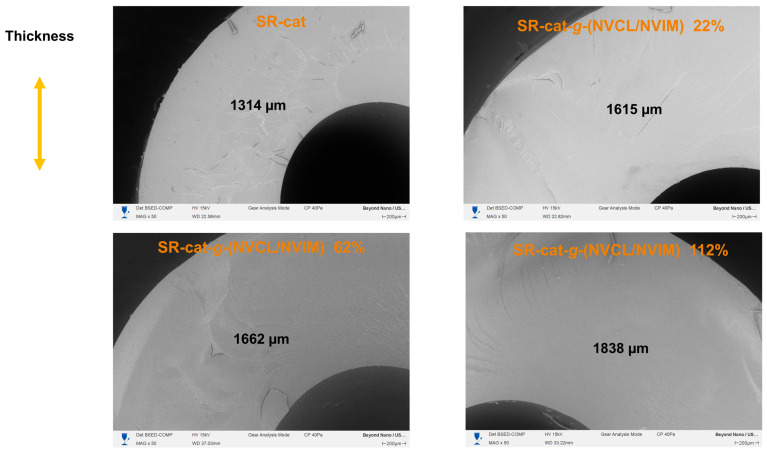
SEM micrographs of the cross-section of SR-cat, SR-cat-*g*-(NVCL/NVIM) 22%, SR-cat-*g*-(NVCL/NVIM) 62% and SR-cat-*g*-(NVCL/NVIM) 112%.

In the cross-sectional analysis, the thickness was measured, and the morphology of the catheters in the cut region was observed. As shown in Figure 9, the thickness of the modified catheters increased compared to that of the silicone catheters. There is a relationship between catheter thickness and graft percentage: as the graft percentage increases, the catheter thickness also increases, confirming that the graft was performed in bulk. When analyzing the topography of the cut region, we observed that the SR-cat-*g*-(NVCL/NVIM) samples showed changes due to grafting.

### 3.10. Mechanical Tests

The mechanical test was conducted on the SR-cat samples and the SR-cat-*g*-(NVCL/NVIM) samples with graft percentages of 25, 60, and 103%. This study aimed to examine the effect of grafting on silicone catheters. Young’s Modulus, stress, and maximum deformation were measured under conditions of a 10 mm/min crosshead speed and a 10 mm distance between the jaws. The tensile test was performed with the swollen specimens, since the purpose of these biomaterials is to apply them while swollen.

Table 1 summarizes the results from this test, showing that SR-cat had an average Young’s modulus of 7.6 MPa and a higher capacity to withstand stresses before fracturing, with an average value of 7.2 MPa, along with a high average deformation of 577.7%. These values indicate a material with high deformation capacity and strong resistance to breakage, aligning with expectations. In contrast, graft copolymers behave differently, exhibiting less flexibility than pure silicone, as reflected in an increased Young’s modulus and decreased elongation or deformation. This behavior can be attributed to gamma radiation-induced cross-linking and to the reduced freedom of polymer chains resulting from the incorporation of grafted monomers into the amorphous regions of the control silicone.

For SR-cat-g-(NVCL/NVIM) 25%, SR-cat-g-(NVCL/NVIM) 60%, and SR-cat-g-(NVCL/NVIM) 103%, a trend is observed: as the percentage of grafting increases, the Young’s modulus and the maximum tensile strength decrease. This is attributed to pre-test swelling; samples with a higher grafting percentage swell more because of the increased number of hydrophilic groups in the material, which can form hydrogen bonds. Therefore, grafted copolymers that swell more are more flexible and exhibit lower tensile strength.

Although the prior swelling favored better results for the grafted copolymers compared to the dry specimens, they remained less flexible than unmodified silicone, as they exhibited higher Young’s Modulus values, lower deformation percentages, and lower stress levels. Therefore, the graft alters the modified catheters, making them less flexible than the original silicone catheters.

### 3.11. Drug Loading and Release

Norfloxacin loading and release were designed to study the material’s capacity to carry the drug and act as a vehicle to transport it to a specific site, allowing for controlled and sustained delivery.

Norfloxacin is a broad-spectrum synthetic antibiotic belonging to the fluoroquinolone family, developed primarily to combat Gram-negative (Gram-) bacteria. This drug is used to treat urinary tract infections [32].

**Figure 10:**
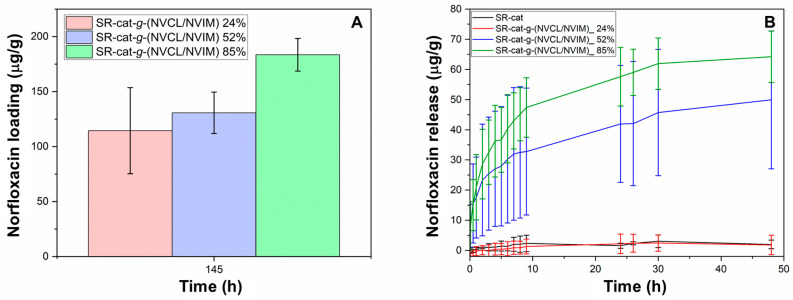
(**A**) Norfloxacin loading profile for SR-cat-*g*-(NVCL/NVIM) 24%, SR-cat-*g*-(NVCL/NVIM) 52%, and SR-cat-*g*-(NVCL/NVIM) 85%. Conditions: 25 °C, initial norfloxacin concentration 8 mg/mL. (**B**) Norfloxacin release profile for SR-cat and SR-cat-*g*-(NVCL/NVIM) samples with different grafting percentages: 24, 52, and 85%. Conditions: pH = 7.4, 37 °C, and stirring at 100 rpm.

Intermolecular interactions, including hydrogen bonding, ionic interactions, and Van der Waals forces, between the drug and the modified polymer matrix enabled norfloxacin loading. Figure 10A shows the results for norfloxacin loading; it can be seen that for the modified catheters, as the graft percentage increases, so does the drug loading (Figure S2). This is due to the increased number of interactions between norfloxacin and the polymer chains. The loading capacity for SR-cat-*g*-(NVCL/NVIM) 24% was 115 mg/mL, for SR-cat-*g*-(NVCL/NVIM) 52% was 131 mg/mL, and for SR-cat-g-(NVCL/NVIM) 85% was 184 mg/mL.

The norfloxacin release assay was conducted for 48 h under the following conditions: PBS (pH 7.4) at 37 °C with agitation at 100 rpm (Figure 10B, Figure S3). The drug release behavior for the modified catheters was: SR-cat-*g*-(NVCL/NVIM) 85% > SR-cat-*g*-(NVCL/NVIM) 52% > SR-cat-*g*-(NVCL/NVIM) 24%, aligning with the amount of drug loaded. After 48 h, not all of the loaded drug had been released; SR-cat-*g*-(NVCL/NVIM) 85% released 64 mg/mL, representing 37% of the loaded drug, while SR-cat-*g*-(NVCL/NVIM) 52% released 50 mg/mL, or 34%. This demonstrates the graft’s influence on norfloxacin release. In contrast, SR-cat-*g*-(NVCL/NVIM) 24% behaved similarly to the control because it had a lower graft percentage, leading to fewer interactions between the drug and the polymer matrix, which, in turn, affected its loading and release behavior.

The norfloxacin release profile was analyzed using Excel Microsoft 365 with DDSolver complement and various mathematical models were applied to determine the best fit to the experimental data (Tables S3 and S4). The study of release kinetics and model selection criteria reveals that the Weibull model is the best fit among the analyzed models. A correlation coefficient of 0.9958 is obtained for SR-cat-*g*-(NVCL/NVIM) 52% and 0.9964 for SR-cat-*g*-(NVCL/NVIM) 85%. This indicates that the Weibull model accurately describes the release profile over 48 h.

## 4. Discussion

*Grafting:* Grafting NVCL/NVIM monomers onto silicone catheters using the direct method and toluene solvent enabled mass grafting with high yields. However, at high irradiation doses, the catheters became stiffer. Therefore, irradiation doses of 40–50 kGy and a monomer concentration of 50% vol in 50% vol toluene are recommended to obtain the SR-cat-*g*-(NVCL/NVIM) copolymer.

*pH and Temperature Response:* The SR-cat-*g*-(NVCL/NVIM) copolymer exhibited a hydrophilic character upon grafting, resulting from the incorporation of hydrophilic groups into its structure. The pH-sensitivity study was performed by swelling the grafted catheters in solutions with pH values ranging from 2 to 12. The critical pH was identified in the range of 8 to 8.5. Although the pH response shifted slightly alkaline values, it is still close to the physiological pH and the pKa of the imidazole group. The LCST was determined at 34 °C, a value consistent with the physiological temperature range. This offers key advantages for biomedical applications, especially in controlled drug delivery systems. The SR-cat-*g*-(NVCL/NVIM) copolymer exhibited a dual response to pH and temperature, which is favorable for drug loading and release.

*Loading and release of norfloxacin:* Norfloxacin, due to its functional groups—ketone, carboxylic acid, and amines—can interact with the modified catheter. Intermolecular interactions between the polymer matrix and norfloxacin facilitated its loading and release. A correlation was found between the grafting percentage and drug release. It was observed that norfloxacin release was enhanced at higher grafting percentages, due to the drug’s increased hydrophilicity. The release was slow and prolonged, which was closely linked to the limit swelling time, which is reached at 12 h. To prevent biofilm formation, local norfloxacin concentrations must be maintained above the Minimum Inhibitory Concentration (MIC) for sufficient time to prevent bacterial adhesion and proliferation. MIC values vary depending on the bacterial species and its resistance; for example, an MIC of 0.83 µg/mL has been reported for *P. aeruginosa* [33]. MIC studies for norfloxacin have also been reported for Gram-positive and Gram-negative bacteria, ranging from 1.25 to 2.57 µg/mL [34]. Therefore, the amounts of norfloxacin released by the SR-cat-g-(NVCL/NVIM) 85% and SR-cat-g-(NVCL/NVIM) 52% systems may be effective in preventing bacterial proliferation; therefore, it is necessary to perform cytotoxicity assays and antimicrobial tests. The Weibull model focuses on identifying a linear relationship between drug release and time, allowing the relationship between drug characteristics in different matrices [35]. The release mechanism has the diffusion model on a fractal or disordered substrate, indicating the occurrence of a release according to Fickian diffusion [36].

## 5. Conclusions

N-vinylcaprolactam and N-vinylimidazole were grafted onto silicone catheters using a direct, single-step irradiation method. Excellent results were achieved with doses between 40 and 50 kGy and a monomer concentration of 50% NVCL/NVIM in toluene. Grafting of NVCL/NVIM enhances swelling and imparts hydrophilicity to the matrix. The synthesized copolymer was sensitive to temperature and pH changes. A phase transition occurred at around 34 °C and a critical pH range of 8 to 8.5. Instrumental analysis confirmed the copolymer grafting, as evidenced by characteristic monomer bands in the FTIR spectrum. Thermal analysis indicated that the graft’s thermal stability was as expected. SEM analysis confirmed the presence of the graft in bulk. The mechanical properties change during the grafting process. The loading and release of norfloxacin were successfully performed, and the release kinetics align with the Weibull model.

## Figures and Tables

**Table 1:** Tensile test results for SR-cat, SR-cat-*g*-(NVCL/NVIM) 25%, SR-cat-*g*-(NVCL/NVIM) 60% and SR-cat-*g*-(NVCL/NVIM) 103%.

Material	Young’s Modulus (MPa)	Maximum Stress (MPa)	Maximum Deformation (%)
SR-cat	7.6 ± 0.9	7.2 ± 0.2	577.7 ± 13.2
SR-cat-*g*-(NVCL/NVIM) 25% graft	101.2 ± 10.5	4.9 ± 0.2	74.2 ± 1.8
SR-cat-*g*-(NVCL/NVIM) 60% graft	19.8 ± 4.3	2.2 ± 0.1	164.4 ± 10.8
SR-cat-*g*-(NVCL/NVIM) 103% graft	15.2 ± 7.5	1.6 ± 0.7	144.5 ± 72.5

## Data Availability

The original contributions presented in the study are included in the article/Supplementary Materials; further inquiries can be directed to the corresponding authors.

## Supplementary Materials

The following supporting information can be downloaded at: https://www.mdpi.com/article/10.3390/polym17233107/s1, Figure S1: Formation of silicone radicals induced by gamma radiation; Figure S2: Calibration curve for norfloxacin loading; Figure S3: Calibration curve for the release of norfloxacin; Table S1: Summary of the contact angle results obtained after 15 min; Table S2: Thermogravimetric analysis results; Table S3: Fitting mathematical models of norfloxacin release SR-cat-*g*-(NVCL/NVIM) 52%; Table S4: Fitting mathematical models of norfloxacin release SR-cat-*g*-(NVCL/NVIM) 85%.

## Abbreviations

The following abbreviations are used in this manuscript:

DSC	Differential scanning calorimetry
FTIR	Fourier-transmittance infrared spectroscopy
LCST	Lower critical solution temperature
NVCL	N-vinylcaprolactam
NVIM	N-vinylimidazole
SEM	Scanning Electron Microscopy
PBS	Phosphate-Buffered Saline
SR-cat	Silicone catheter
Tg	Glass transition temperature
TGA	Gravimetric thermal analysis
