# Supplementary material for: Synthesis of a Stimulus-Sensitive Copolymer with Response to Temperature and pH in Silicone Catheters
Source: Polymers (Basel). 2025 Nov 23;17(23):3107. doi: 10.3390/polym17233107 (PMC12694089; doi:10.3390/polym17233107)
Supplement: Supplementary file 1 [file polymers-17-03107-s001.zip › polymers-3950643-supplementary.pdf]

polymers

Supplementary Materials

Synthesis of a Stimulus-Sensitive Copolymer with Response to Temperature and pH in Silicone Catheters

Yanet González Alonso * and Emilio Bucio *

Departamento de Química de Radioquímica, Instituto de Ciencias Nucleares, Universidad Nacional Autónoma de México, Circuito Exterior, Ciudad Universitaria, Mexico City C.P. 04510, Mexico
* Correspondence: yanet.gonzalez@correo.nucleares.unam.mx (Y.G.A.); ebucio@nucleares.unam.mx (E.B.)

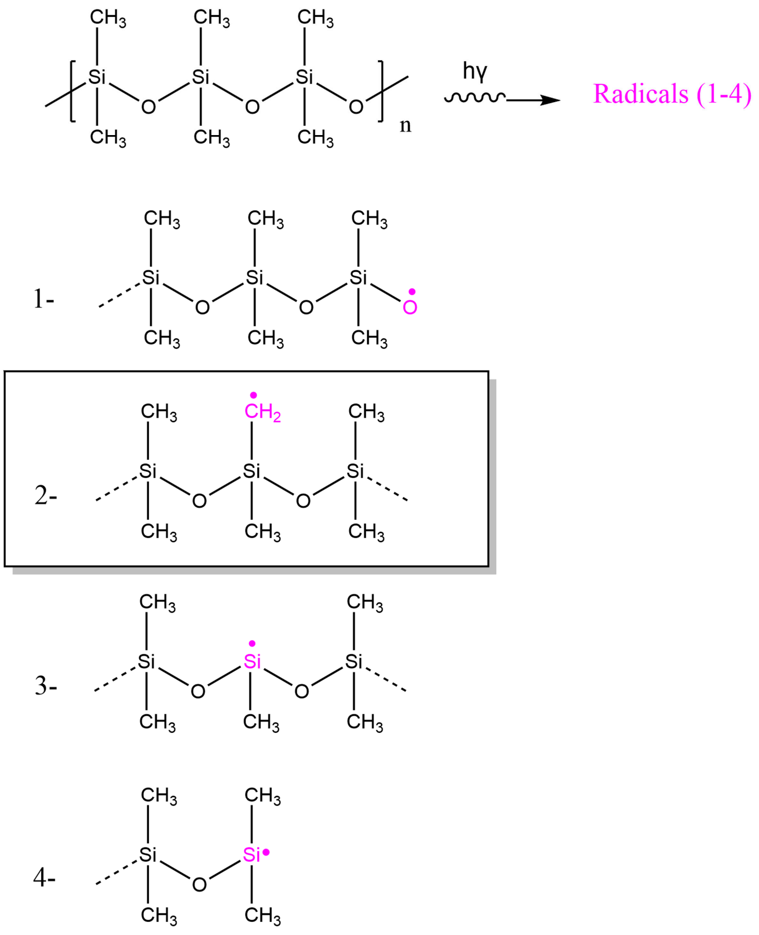

Figure S1. Formation of silicone radicals induced by gamma radiation.

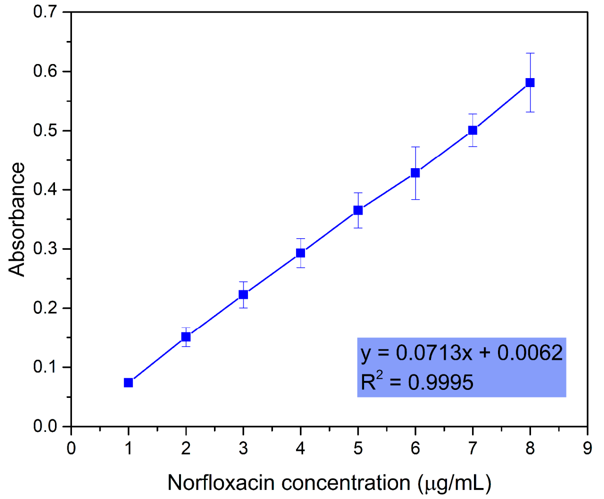

Figure S2. Calibration curve for norfloxacin loading.

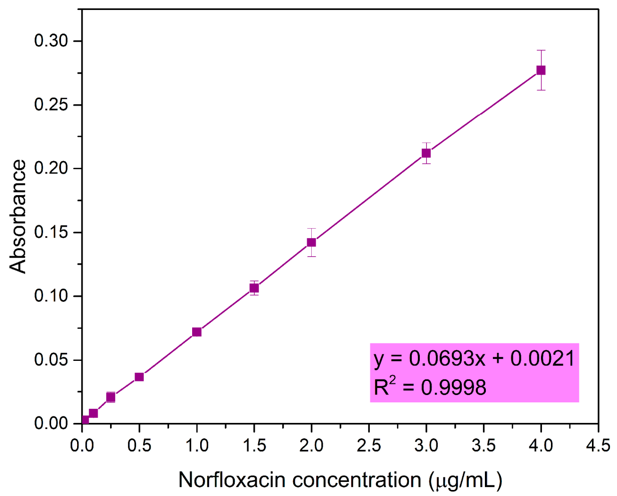

Figure S3. Calibration curve for the release of norfloxacin.

Table S1. Summary of the contact angle results obtained after 15 minutes.

Material	Contact Angle (°)	Drop Image
SR-cat	87 ± 1.1	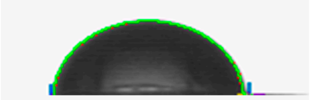
SR-cat-*g*-(NVCL/NVIM) 28%	69 ± 4	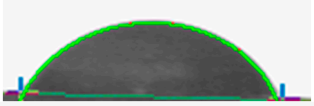
SR-cat-*g*-(NVCL/NVIM) 79%	54 ± 2.3	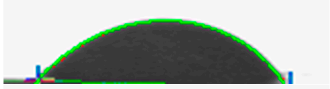
SR-cat-*g*-(NVCL/NVIM) 90%	41 ± 11.1	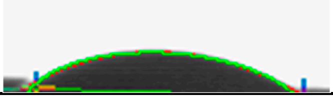

Table S2. Thermogravimetric analysis results.

Material	10 % Weight loss (˚C)	Descomposition temperature (˚C)	Residue @ 800 ˚C (%)
SR-cat	547	533<br>702	66 %
SR-cat-*g*-(NVCL/NVIM) 18%	474	473<br>632	35 %
SR-cat-*g*-(NVCL/NVIM) 47%	455	472<br>637	27 %
SR-cat-*g*-(NVCL/NVIM) 92%	452	476<br>643	25 %
Copolymer NVCL/NVIM	261	439	9 %

Table S3. Fitting mathematical models of norfloxacin release. SR-cat-*g*-(NVCL/NVIM) 52%.

Models	R^2	AIC	MSC
First-order with Tlag and Fmax	0.9795	57.7087	3.0713
Peppas-Sahlin 1 with Tlag	0.9940	43.3904	4.0259
Korsmeyer-Peppas with F0	0.9934	42.5328	4.0830
Weibull	0.9958	38.5669	4.1392
Logistic	0.9954	38.9672	4.3207
Gompertz	0.9950	39.1292	4.3100
Probit	0.9952	39.5364	4.2828
Makoid-Banakar with Tlag	0.9955	39.6521	4.2751

Table S4. Fitting mathematical models of norfloxacin release. SR-cat-*g*-(NVCL/NVIM) 85%.

Models	R^2	AIC	MSC
First-order with Tlag and Fmax	0.9884	53.0799	3.5284
Peppas-Sahlin 1 with Tlag	0.9956	42.9170	4.2060
Korsmeyer-Peppas with F0	0.9918	49.8451	3.7441
Weibull	0.9964	39.1561	4.4567
Logistic	0.9958	38.3311	4.5117
Gompertz	0.9952	40.5649	4.3628
Probit	0.9955	39.1517	4.4570
Makoid-Banakar with Tlag	0.9956	42.7304	4.2184
